# Supplementary material for: The Antithrombotic Function of Sphingosine-1-Phosphate on Human Adipose-Stem-Cell-Recellularized Tissue Engineered Vascular Graft In Vitro
Source: Int J Mol Sci. 2019 Oct 21;20(20):5218. doi: 10.3390/ijms20205218 (PMC6829437; doi:10.3390/ijms20205218)
Supplement: Supplementary file 1 [file ijms-20-05218-s001.pdf]

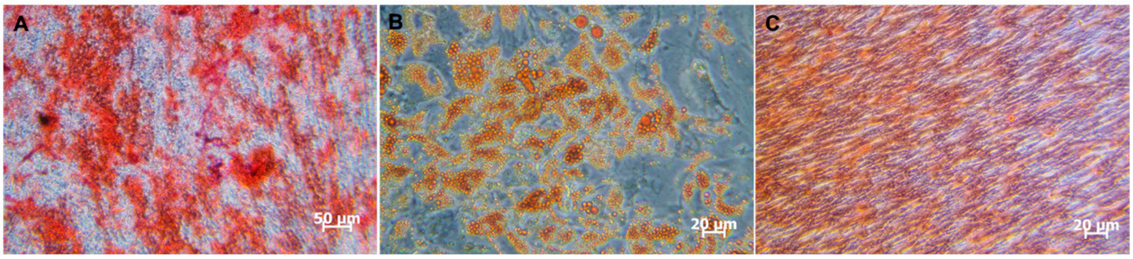

Figure S1. Osteogenic, Adipogenic, and chondrogenic potential of hASCs. hASCs were induced to differentiate toward the three mesodermal lineages. **A**: osteogenesis: calcium deposits visualized by Alizarin Red staining; **B**: adipogenesis: lipid content stained by Oil Red O staining; **C**: chondrogenesis: Safranin O Staining.

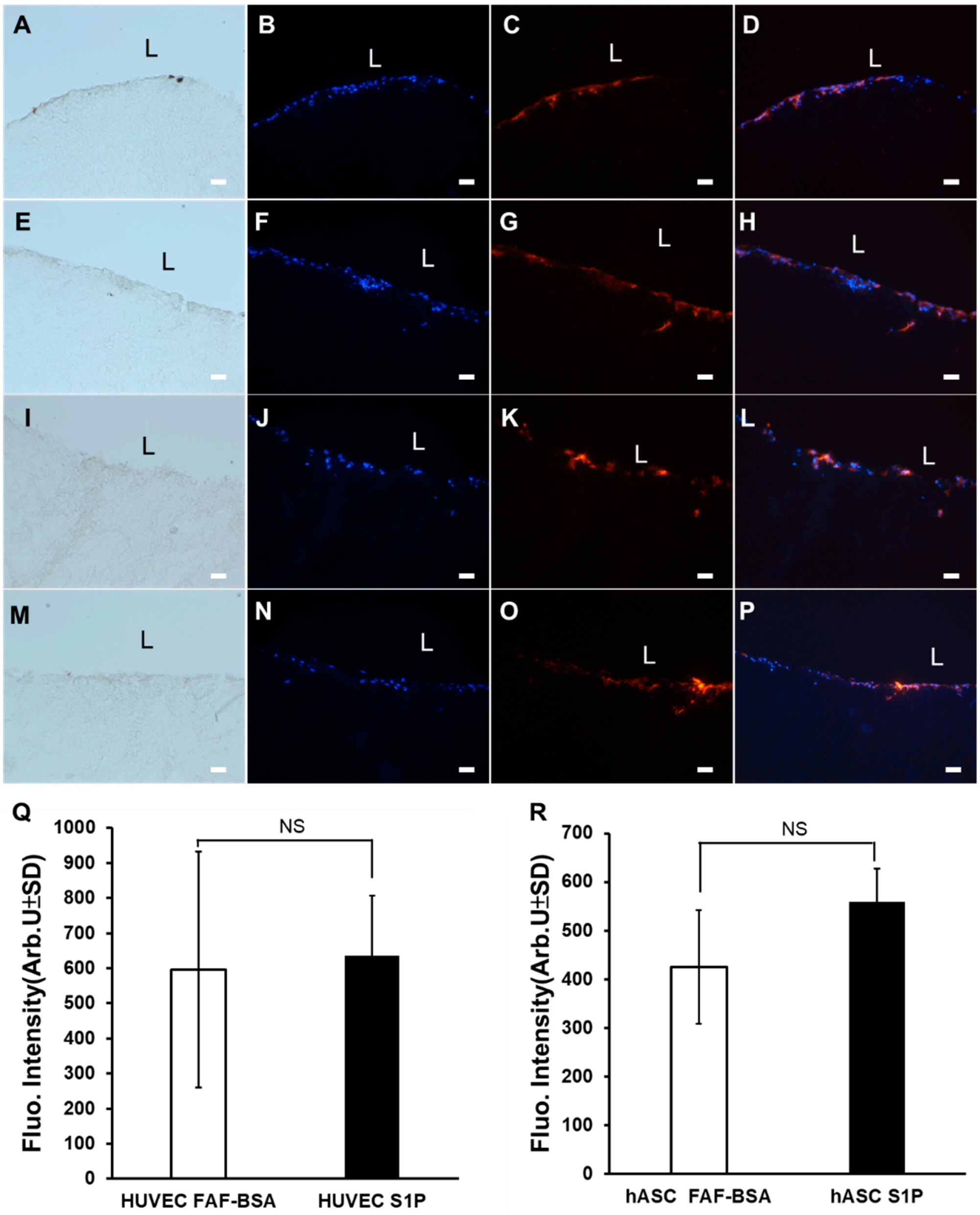

Figure S2. Adhesion and proliferation of hASC and HUVEC on DHUA with S1P at day 14**.** Cells prestained by CM-Dil seeded on DHUA under 14 days of S1P treatment. first column: bright field, second column: CM-Dil, third column: DAPI, fourth column: merge. **A**,**B**,**C**,**D**: images of HUVECs

seeded on DHUA without S1P; **E**,**F**,**G**,**H**: images of HUVECs seeded on DHUA with S1P; **I**,**J**,**K**,**L**: images of hASCs seeded on DHUA without S1P; **M**,**N**,**O**,**P**: images of hASCs seeded on DHUA with S1P; **Q**: fluorescence intensity of CM-Dil in HUVEC-seeded DHUA without and with S1P; **R**: fluorescence intensity of CM-Dil in hASC-seeded DHUA without and with S1P. It indicated that both HUVEC and hASC reached about 80% coverage to the DHUV and there was no significantly difference between the groups in present S1P and without S1P when the treatment was continuous to day 14, (all magnification 200×, scale bar = 5 μm). L: Lumen. NS: not significant.
